# Development and validation of the Patient Involvement in Medication Communication at Hospital discharge Questionnaire (PIMCH-Q)

**DOI:** 10.1186/s41687-026-01136-8

**Published:** 2026-06-29

**Authors:** Victoria Östman, Emma Bertilsson, Henrik Cam, Kristin Franzon, Sofia Kälvemark Sporrong, Emelie Frödén, Sham Shaat, Ulrika Gillespie

**Affiliations:** 1 Department of Pharmacy, Uppsala University, Uppsala, Sweden; 2 Pharmacy Department, Uppsala University Hospital, Uppsala, Sweden; 3 Department of Public Health and Caring Sciences, Uppsala University, Uppsala, Sweden

## Abstract

**Background:**

Medication-related problems are common after hospital discharge, often due to inadequate communication about changes made to medication therapy. Patients’ ability to understand and manage medication changes after discharge is essential for safe and effective treatment. However, existing patient-reported outcome measures (PROMs) do not adequately capture how patients perceive their involvement or understanding of peri- and post-discharge medication communication. The aim of this study was to develop the Patient Involvement in Medication Communication at Hospital discharge Questionnaire (PIMCH-Q) and to evaluate its content validity, structural validity, and internal consistency.

**Methods:**

The 8-item PIMCH-Q was developed based on literature and expert input. Content validity was assessed through expert panel review and patient interviews. Structural validity was evaluated using exploratory and confirmatory factor analyses (EFA, CFA) based on responses from recently discharged patients (n=57). Internal consistency was assessed using McDonald’s omega.

**Results:**

Eleven patients participated in interviews. They found the questionnaire relevant and easy to understand. Exploratory factor analysis suggested a three-factor structure representing perceived involvement in medication decisions, knowledge and understanding of medication changes, and confidence in medication self-management. One item did not show a clear primary loading and was excluded, resulting in a seven-item factor structure. Confirmatory factor analysis demonstrated good model fit (χ²(11) = 6.58; CFI = 1.00; TLI = 1.01; RMSEA = 0.00; SRMR = 0.055). Internal consistency was high (ω total = 0.95).

**Conclusions:**

This study provides preliminary support for the validity and internal consistency of the PIMCH-Q as a PROM for assessing patient experiences of medication communication at hospital discharge. The findings also suggest that the PIMCH-Q may be useful in both research and clinical practice. Further validation in larger and independent samples is warranted.

**Supplementary Information:**

The online version contains supplementary material available at 10.1186/s41687-026-01136-8.

## Introduction

Medication-related problems (MRPs) after discharge may lead to adverse drug events, reduced adherence, treatment failure, emergency visits, and hospital readmissions [1, 2]. Older adults with multimorbidity and polypharmacy are particularly vulnerable, as they often face multiple medication changes and complex follow-up needs during transitions of care [3–5]. In Sweden, legislation requires that medication changes persisting after discharge be accurately documented in the discharge summary and clearly communicated to both the patient and their next healthcare provider [6, 7]. Despite these requirements, studies indicate persistent gaps in written discharge documentation quality and limited patient understanding of medication changes [5, 8, 9].

Patient involvement is associated with improved adherence, satisfaction, and clinical outcomes [6, 10–12]. To evaluate improvements in medication communication, reliable measures from the patient perspective are essential. Furthermore, standardized patient-reported outcome measures (PROMs) enable health care systems to track performance, support improvement, and align with regulation [7].

Few validated instruments exist that assess how patients perceive discharge communication, specifically in relation to their own confidence in managing medications [13–15]. To our knowledge, no validated PROM specifically evaluates patients’ perceived understanding, involvement, and confidence in medication self-management in the context of discharge medication communication [14].

The Patient Involvement in Medication Communication at Hospital discharge Questionnaire (PIMCH-Q) was developed to address this gap. The questionnaire was developed within the context of the *Improved Medication communication and Patient Involvement at Care Transitions (IMPACT-care)* study [16]. The IMPACT-care intervention includes a patient brochure with suggested questions to support active participation during the discharge consultation, preparation of medication-related discharge documentation by clinical pharmacists summarizing medication changes, and a structured follow-up telephone call within one week after discharge to review the medication regimen in a calmer setting. These components aim to strengthen patients’ understanding of medication changes, involvement in medication-related decisions, and their confidence in managing medications after discharge. To evaluate the effectiveness of this intervention from the patient perspective, a dedicated PROM was required. Consequently, the PIMCH-Q was developed as a secondary outcome measure in the study.

The aim of this study was to develop PIMCH-Q and to evaluate its content validity, structural validity, and internal consistency.

## Methods

### Study design

This study used a multiple-method design, combining content validation via expert review and patient interviews with structural validation via factor analysis. The study was inspired by the COnsensus-based Standards for the selection of health Measurement INstruments (COSMIN) guidelines for PROM validation [17].

### Study setting and participants

Patients were recruited from the ongoing IMPACT-care study [16], conducted at Uppsala University Hospital in Sweden. Eligible participants for the IMPACT-care study were aged 65 years or older, managed their own medications prior to admission, experienced at least one medication change that persisted after discharge, and completed PIMCH-Q after leaving the hospital. Exclusion criteria included cognitive impairment, non-Swedish speakers, hospital stays shorter than 48 h, admission to the transplant surgery unit, transition to palliative care or discharge to a nursing facility. All participants provided informed consent prior to study participation.

### Development of the PIMCH-Q

The PIMCH-Q was developed based on literature [18–20] and expert input. The development of the instrument drew on prior instruments conceptually, though no items were adopted verbatim. Item development was informed by constructs identified in previous patient-reported instruments related to medication burden, medication safety involvement, and beliefs about medicines, including the Medication-Related Burden Quality of Life tool (MRB-QoL) [18], the Inpatients’ Involvement in Medication Safety Scale (IIMSS) [19], and the Beliefs about Medicines Questionnaire (BMQ) [20]. In addition, items were developed to reflect the specific components of the IMPACT-care intervention, aiming to capture changes in patient involvement, understanding of medication changes, and confidence in medication self-management targeted by the intervention.

Draft items were generated to reflect three intended content areas: understanding of medication changes, involvement in medication-related decisions, and confidence in managing medications after discharge. These draft items were then refined through expert panel review and patient interviews.

The initial draft version of the PIMCH-Q included eight items, which were subsequently refined based on expert feedback and factor analytic results. The items are rated on a four-point Likert scale ranging from 1 to 4 (“I strongly disagree” to “I strongly agree”). An additional “Don’t know” option was included for items where relevant (item 1–3) and was treated as missing data in the analyses. Higher item scores indicate greater agreement with the statement being assessed.

### Content validation

#### Expert panel reviews

The expert panel comprised six pharmacists, a physician, a nurse, two public representatives and a professor in social pharmacy. The two public representatives were involved in the IMPACT-care research team throughout the intervention development process: CB, who holds political duties advocating for patients, and UE, who serves as the chairperson of an association for relatives of older patients.

Members reviewed items for relevance, clarity, and response options. Panel members were informed about the overall purpose of the PROM and its intended use in the IMPACT-care project. However, they were not given specific instructions to identify domains or themes within the instrument, allowing feedback to focus on the perceived clarity, relevance, and comprehensiveness of the items. Feedback was collected in writing and thereafter discussed in a joint meeting.

#### Patient interviews

Semi-structured interviews were conducted by telephone with patients who had recently been discharged from hospital and had completed the PIMCH-Q as part of the IMPACT-care study. The interviewed patients were recruited from the same study population as those included in the psychometric analyses. Patients were recruited consecutively from the study cohort and invited to participate in an interview exploring their perceptions of the questionnaire. To capture variation within the control period, recruitment was conducted during two time windows: one at the beginning of the control period (September, 1 month) and one at the end of the control period (February, 1 month). The control period refers to the pre-intervention phase of the IMPACT-care study, during which usual care was provided without the intervention components.

A cognitive interviewing approach using the think-aloud method was applied, supported by a semi-structured interview guide to ensure that key topics were addressed while allowing participants to elaborate freely.

The interview guide focused on item clarity, relevance, and comprehensiveness. Each questionnaire item was read aloud by the interviewer and followed by standardized open-ended probes. These probes explored (1) clarity and comprehension (e.g., “Was this question easy or difficult to understand?”) (2), interpretation (e.g., “What did this question mean to you?” and “What were you thinking about when answering?”), and (3) relevance (e.g., “Do you consider this question important for your experience?”). Participants were encouraged to elaborate freely and provide examples where applicable. Interviews were audio-recorded, transcribed verbatim, and analyzed using a descriptive approach.

Interviews were conducted until data saturation was considered to have been reached, meaning that no new issues related to item clarity or relevance emerged in subsequent interviews.

### Structural validity and internal consistency

Psychometric properties were evaluated in 57 patients. A complete-case approach was used for the psychometric analyses, and only respondents with complete PIMCH-Q data were included. These patients were participants in the ongoing IMPACT-care study and completed the questionnaire as part of that study. At the time of data extraction for this analysis, 57 patients had fully completed the PIMCH-Q and were therefore included in the psychometric analyses. Both EFA and CFA were conducted on the same complete-case sample. The underlying factor structure was first explored using exploratory factor analysis (EFA) based on a polychoric correlation matrix, with minimum residual extraction and oblimin rotation to allow correlated factors. Based on the EFA results, a confirmatory factor analysis (CFA) was conducted to assess model fit [21, 22]. Items that did not demonstrate a clear primary loading in the EFA were excluded from the CFA. Model performance was evaluated using Comparative Fit Index (CFI), Tucker-Lewis Index (TLI), Root Mean Square Error of Approximation (RMSEA), and Standardized Root Mean Square Residual (SRMR). Internal consistency was assessed using McDonald’s omega [23, 24]. All statistical analyses were performed in RStudio [25].

### Ethical considerations

The IMPACT-care study has been approved by the Swedish Ethical Review Authority (registration no: 2023-03518-01 and 2024-04079-02).

## Results

### Content validation

#### Expert panel reviews

Based on recommendations from the expert panel, response options were revised in accordance with the Statistics Sweden (SCB) [26] guidelines for survey construction to ensure consistency with standard Swedish linguistic usage and improve clarity. In the first draft of the questionnaire, items 3 and 4 were combined into a single question. The expert panel emphasized the importance of distinguishing between “what” and “why” and therefore recommended splitting the item into two separate items (Table 2).

#### Patient interviews

Eleven patients participated in interviews and overall found the PIMCH-Q relevant and easy to understand. Items were described as reflective of their discharge experience, particularly in areas typically overlooked, such as knowing where to turn with questions. Some patients had difficulty distinguishing between item 3 and item 4 (Table 2), the rationale (“why”) and specifics (“what”) of medication changes, but found both items acceptable when clarified. To improve clarity, the terms “why” and “what” were subsequently highlighted in bold in the questionnaire. Both items were retained in the final version. The inclusion of a “don’t know” response option was appreciated, especially for memory-dependent items related to the hospital stay.

### Psychometric testing

#### Patient characteristics

Patient characteristics for the psychometric sample (*n* = 57) are presented in Table 1.

**Table 1:** Patient characteristics (*n* = 57)

**Age, years**	77 (72–82)
**Sex**	
Female	35 (61.4%)
Male	22 (38.6%)
**Length of stay at study ward**,** days**	6 (4–8)
**Number of medications at admission**	7.5 (4–11)
**Number of medications at discharge**	9.5 (7–15)
**Total number medication changes**	4 (2–5)
**Social support**	
Spouse/partner	34 (59.6%)
Home care	5 (8.8%)
None	18 (31.6%)
**Highest completed education**	
Elementary school	14 (24.6%)
Upper secondary school	16 (28.1%)
University/College	26 (45.6%)
**Opt out / missing data**	1 (1.8%)

Values are presented as median (IQR) or n (%)

#### Exploratory factor analysis

The Kaiser-Meyer-Olkin measure for sampling adequacy was 0.73 indicating good suitability of the data for factor analysis. Parallel analysis suggested a three-factor solution, which was supported by the eigenvalues of the polychoric correlation matrix (4.47, 1.14, and 1.02 for the first three factors). Exploratory factor analysis using minimum residual extraction and oblimin rotation supported a three-factor structure. The original item 2, “I was offered the opportunity to have an informal caregiver present during the discharge consultation”, did not show a clear primary loading in the retained three-factor solution and was therefore excluded from the confirmatory factor analysis. It was conceptually also considered a process-related question. The remaining seven items were grouped into three domains: involvement, knowledge, and confidence (Table 2). The full questionnaire is available in Supplementary File 1.

**Table 2:** Overview of PIMCH-Q thematic domains and corresponding items. This table lists the three thematic domains identified through factor analysis and their corresponding items in the PIMCH-Q questionnaire

Domain	Item
Involvement (Factor 1)	1. I felt involved in decisions about my medication treatment that would continue after discharge (e.g., which changes would be made).
	3. I felt involved in decisions about the follow-up of my medication treatment.
Knowledge (Factor 2)	4. I (and/or my informal caregiver) know what changes were made to my medication treatment in the hospital (e.g., new medications, medications I should no longer use, or changes in dosage).
	5. I (and/or my informal caregiver) know why my medication treatment was changed in the hospital (e.g., due to newly discovered atrial fibrillation or high blood pressure).
Confidence (Factor 3)	6. I feel confident that I (and/or informal caregiver) can manage my medication treatment.
	7. I (and/or informal caregiver) know where to turn if I have questions about my medication treatment.
	8. I (and/or my relative) know how my medication treatment will be followed up.

Primary factor loadings in the three-factor solution ranged from 0.60 to 1.02 (Table 3). Item 2 did not load > 0.40 on any factor. Factor correlations ranged from 0.45 to 0.54, indicating related but distinct constructs (Table 4).

**Table 3:** Exploratory factor analysis results: factor loadings and interpretation

Item	Factor 1	Factor 2	Factor 3	Complexity
1	0.83			1.0
3	1.00			1.0
4		1.02		1.0
5		0.67		1.1
6			0.60	1.5
7			1.02	1.0
8			0.88	1.1
2				1.8

Only loadings > 0.40 are displayed

**Table 4:** Correlations among latent factors

Factor	Factor 1	Factor 2	Factor 3
Factor 1	1.00	0.54	0.48
Factor 2	0.54	1.00	0.45
Factor 3	0.48	0.45	1.00

#### Confirmatory factor analysis

Confirmatory factor analysis showed that the three-factor model provided substantially better fit than the one-factor model. The one-factor model demonstrated poor fit (χ² (14) = 83.29; CFI = 0.95; TLI = 0.93; RMSEA = 0.30; SRMR = 0.17). In contrast, the three-factor model demonstrated good model fit (χ² (11) = 6.58; CFI = 1.00; TLI = 1.01; RMSEA = 0.00; SRMR = 0.055). Standardized factor loadings were strong, ranging from 0.81 to 1.03 for Factor 1, 0.83 to 1.01 for Factor 2, and 0.86 to 0.91 for Factor 3. Correlations among latent factors ranged from 0.56 to 0.59, indicating moderate associations between the constructs. Due to the limited sample size, the confirmatory factor analysis was conducted on the same dataset as the exploratory factor analysis. Two standardized loadings slightly exceeded 1.0.

#### Internal consistency

Missing data were low (< 5% per item). McDonald’s Omega total was 0.95, Omega hierarchical 0.69, and explained common variance (ECV) was 0.52, supporting overall reliability while indicating multidimensionality of the identified factor structure.

## Discussion

The aim of this study was to develop the Patient Involvement in Medication Communication at Hospital discharge Questionnaire (PIMCH-Q) and to evaluate its content validity, structural validity, and internal consistency. Overall, the findings provide preliminary evidence that the instrument is relevant to patients, acceptable in terms of content and clarity, and characterized by a three-factor structure reflecting involvement, knowledge, and confidence.

These domains are highly relevant in the context of hospital discharge, where medication regimens are often changed and responsibility for treatment management shifts from healthcare professionals to patients and informal caregivers [27, 28]. Knowledge reflects whether patients understand what medication changes were made and why. Involvement reflects whether they perceive that they were included in decisions regarding their medication treatment and its follow-up. Confidence reflects whether they feel able to manage the treatment after discharge, know where to turn with questions, and understand how follow-up will occur. Together, these domains represent central aspects of person-centred and safe medication communication during care transitions.

These findings align with growing patient-centred care priorities, which emphasize active patient participation, shared decision-making, and effective communication across care transitions. Previous work has highlighted that patient-centred communication is associated with improved health outcomes, and patient experiences, partly through enhanced patient understanding and health competence [27]. Research on hospital-to-home transitions has emphasized the importance of patient and caregiver involvement, as well as coordination and adequate support during the transition from hospital to home, where patients often experience unmet needs and fragmented care [28]. The domains identified in the PIMCH-Q therefore correspond well to issues already recognized as important in transitional care, while offering a structured patient-reported way to assess them.

A recent scoping review identified key domains of patient-reported experience across healthcare settings, including communication, coordination, and person-centred aspects of care [29]. The domains identified in this study, knowledge, involvement, and confidence map closely onto these broader constructs, but with a specific focus on medication communication at discharge. This is a clinically relevant distinction, as medication-related problems after discharge often arise not only from documentation errors or incomplete medication lists, but also from communication failures and patient-level factors such as limited understanding, low health literacy, insufficient involvement, and uncertainty regarding medication management at home [30–33].

From a clinical perspective, the PIMCH-Q may be useful for identifying gaps in discharge communication that are not captured by traditional clinical or documentation-based measures. For instance, low scores in the knowledge domain may indicate insufficient explanation of medication changes, while low confidence scores may signal a need for additional counselling or closer follow-up after discharge. Low involvement scores may indicate that patients were not adequately included in treatment decisions or follow-up planning. In this way, the questionnaire may help identify specific areas in discharge routines that require improvement.

The PIMCH-Q may also be useful in research and quality improvement. In intervention studies such as IMPACT-care, it could be used to evaluate whether structured discharge processes improve patient-perceived understanding, involvement, and self-management capacity [16]. In routine practice, repeated use of the questionnaire could support local monitoring of patient experiences over time and help evaluate whether changes in discharge routines lead to measurable improvements. The findings of this study therefore suggest that the PIMCH-Q may complement traditional outcomes such as readmissions or medication-related problems by adding insight into patient-level mechanisms that may contribute to safer or less safe transitions.

### Strengths and limitations

A key strength of this study is the rigorous and iterative development process of the questionnaire, incorporating multiple sources of input, including literature, expert panel evaluation, patient interviews, and psychometric testing within the target population. This approach ensured both conceptual relevance and practical applicability. The expert panel’s emphasis on distinguishing between *what* medication changes were made and *why* they were made, together with patient feedback supporting the inclusion of a “don’t know” response option, contributed to improved clarity and interpretability of the items. Furthermore, internal consistency was assessed using McDonald’s omega rather than Cronbach’s alpha, as omega is more appropriate when assumptions of equal item loadings are unlikely to be met, particularly in multidimensional instruments such as the PIMCH-Q.

However, the findings should be interpreted in light of several limitations. First, the study was conducted at a single university hospital in Sweden, limiting generalizability. Second, the psychometric analyses were based on a small complete-case sample (*n* = 57), and the confirmatory factor analysis was performed on the same dataset as the exploratory factor analysis because the sample was too small to permit a split-sample or independent validation approach. This increases the risk of overfitting and means that the CFA should be interpreted as a preliminary assessment of model fit rather than an independent confirmation of the factor structure. Third, the CFA showed minor estimation problems, including standardized loadings slightly above 1.0 and negative residual variances for two items, indicating possible Heywood cases [34]. These findings likely reflect the small sample size and the presence of factors with only two indicators and reinforce the need for replication in larger and more diverse samples. The patient interview sample was relatively small (*n* = 11). However, interviews were conducted until data saturation was reached, suggesting that the main issues related to item clarity and relevance were adequately captured.

Despite these limitations, the findings provide preliminary support for the validity and potential utility of the instrument.

## Conclusion

This study provides preliminary support for the validity and internal consistency of the PIMCH-Q as a patient-reported outcome measure for assessing patient experiences of medication communication at hospital discharge. The questionnaire captures key domains of involvement, knowledge, and confidence that are relevant to patient-centred care and medication safety.

The findings also suggest that the PIMCH-Q may be useful in both research and clinical practice. It has the potential to support routine monitoring of patient experiences, identify gaps in discharge communication, and inform the design of safer and more patient-centred discharge processes. Future research should evaluate the factor structure in a larger independent sample, examine test-retest reliability and responsiveness, and assess its utility as part of larger interventions aimed at reducing post-discharge medication-related harm.

## Supplementary Information

Below is the link to the electronic supplementary material.

Supplementary Material 1

## Data Availability

The datasets used and/or analysed during the current study are available from the corresponding author on reasonable request.

## Abbreviations

BMQ: Beliefs about Medicines Questionnaire
CFI: Comparative Fit Index
COSMIN: COnsensus–based Standards for the selection of health Measurement INstruments
CFA: Confirmatory Factor Analysis
ECV: Explained Common Variance
EFA: Exploratory Factor Analysis
IIMSS: Inpatients’ Involvement in Medication Safety Scale
IMPACT: care–Improved Medication Communication and Patient Involvement at Care Transitions
IQR: Interquartile Range
KMO: Kaiser–Meyer–Olkin
MRB: QoL–Medication–Related Burden Quality of Life tool
MRP: Medication–Related Problem
PIMCH: Q–Patient Involvement in Medication Communication at Hospital discharge Questionnaire
PROM(s): Patient–Reported Outcome Measure(s)
RMSEA: Root Mean Square Error of Approximation
SCB: Statistics Sweden
SRMR: Standardized Root Mean Square Residual
TLI: Tucker–Lewis Index
