# Supplementary material for: Development and validation of the Patient Involvement in Medication Communication at Hospital discharge Questionnaire (PIMCH-Q)
Source: J Patient Rep Outcomes. 2026 Jun 29;10:107. doi: 10.1186/s41687-026-01136-8 (PMC13319526; doi:10.1186/s41687-026-01136-8)
Supplement: Supplementary file 1 — Supplementary Material 1 [file 41687_2026_1136_MOESM1_ESM.docx]

**Patient Involvement in Medication Communication at Hospital discharge Questionnaire (PIMCH-Q)**

(Unofficial English version, translated from Swedish)

| **Items** | I strongly disagree | I disagree | I agree | I strongly agree | Don’t know |
| --- | --- | --- | --- | --- | --- |
| **While in hospital…** |  |  |  |  |  |
| 1. I felt involved in decisions about my medication treatment that would continue after discharge (e.g., which changes would be made). |  |  |  |  |  |
| 2. I was offered the opportunity to have an informal caregiver present during the discharge consultation. |  |  |  |  |  |
| 3. I felt involved in decisions about the follow-up of my medication treatment. |  |  |  |  |  |
| **After returning home…** |  |  |  |  |  |
| 4. I (and/or my informal caregiver) know what changes were made to my medication treatment in the hospital (e.g., new medications, medications I should no longer use, or changes in dosage). |  |  |  |  |  |
| 5. I (and/or my informal caregiver) know why my medication treatment was changed in the hospital (e.g., due to newly discovered atrial fibrillation or high blood pressure). |  |  |  |  |  |
| 6. I feel confident that I (and/or informal caregiver) can manage my medication treatment. |  |  |  |  |  |
| 7. I (and/or informal caregiver) know where to turn if I have questions about my medication treatment. |  |  |  |  |  |
| 8. I (and/or my relative) know how my medication treatment will be followed up. |  |  |  |  |  |
